# The Roles of Neurotrophins in Traumatic Brain Injury

**DOI:** 10.3390/life12010026

**Published:** 2021-12-24

**Authors:** Ping-Hung Lin, Lu-Ting Kuo, Hui-Tzung Luh

**Affiliations:** 1 Department of Medical Education, School of Medicine, National Taiwan University, Taipei 100, Taiwan; b03401063@ntu.edu.tw; 2 Division of Neurosurgery, Department of Surgery, National Taiwan University Hospital, Taipei 100, Taiwan; ltkuo@ntu.edu.tw; 3 Department of Neurosurgery, Shuang Ho Hospital, Taipei Medical University, New Taipei City 235, Taiwan; 4 Taipei Neuroscience Institute, Taipei Medical University, New Taipei City 235, Taiwan; 5 Graduate Institute of Clinical Medicine, National Taiwan University, Taipei 100, Taiwan

**Keywords:** neurotrophins, traumatic brain injury, nerve growth factor, brain-derived neurotrophic factor, NT-3, NT-4/5

## Abstract

Neurotrophins are a collection of structurally and functionally related proteins. They play important roles in many aspects of neural development, survival, and plasticity. Traumatic brain injury (TBI) leads to different levels of central nervous tissue destruction and cellular repair through various compensatory mechanisms promoted by the injured brain. Many studies have shown that neurotrophins are key modulators of neuroinflammation, apoptosis, blood–brain barrier permeability, memory capacity, and neurite regeneration. The expression of neurotrophins following TBI is affected by the severity of injury, genetic polymorphism, and different post-traumatic time points. Emerging research is focused on the potential therapeutic applications of neurotrophins in managing TBI. We conducted a comprehensive review by organizing the studies that demonstrate the role of neurotrophins in the management of TBI.

## 1. Biochemistry of Neurotrophins

Neurotrophins are a collection of structurally and functionally related proteins. They undergo proteolytic processes from proneurotrophins (precursor proteins) that possess a C-terminal mature domain and an N-terminal prodomain [1]. The prodomains ensure proper protein folding and dimerization, whereas the mature domains play a role in the biological effects of neurotrophins [2]. They were first discovered as physiological regulators of the sympathetic and sensory neurons that modulate neuronal survival, function, and development not only within the peripheral nervous system (PNS) but also in the central nervous system (CNS) [3]. These factors control axonal plasticity, neuronal survival, and several synaptic functions, including neurotransmitter availability [4]. Many neurotrophic factors such as the basic fibroblast growth factor (bFGF), ciliary neurotrophic factor (CNTF), glial cell line-derived neurotrophic factor (GDNF), insulin growth factors (IGFs), transforming growth factors (TGFs), and tumor necrosis factors (TNFs) can attenuate neuronal injury and regulate the differentiation, survival, and maintenance of nerve cells [5]. However, the term “neurotrophin” is more commonly used to refer to four structurally related biomolecules only. In this review, we will primarily focus on the characteristics of the four neurotrophins in the human body: nerve growth factor (NGF), brain-derived neurotrophic factor (BDNF), neurotrophin 3 (NT-3), and neurotrophin 4 (NT-4), sometimes known as neurotrophin 5 (NT-5) (NT-4/5) [6].

Neurotrophins can bind to two separate classes of receptors: p75 neurotrophin receptor (p75^NTR^) and tropomyosin receptor kinase (Trk) receptors. All members of the mature neurotrophin family have a lower affinity for the p75^NTR^ receptor than proneurotrophins [7]. Meanwhile, individual neurotrophins activate corresponding Trk receptors with high specificity and affinity, with NGF binding to TrkA, BDNF and NT-4/5 binding to TrkB, and NT-3 binding to TrkC [8,9,10]. TrkB binds BDNF and NT-4/5 more strongly than NT-3. TrkC binds NT-3 more tightly than TrkB [11]. Furthermore, Verdi et al. showed that p75^NTR^ augments the response of TrkA to NGF [12,13]. In fact, Ip et al. discovered that a neuronal environment limits the ability of Trk receptors to bind to their specifically non-preferred neurotrophin ligands [14].

The complex interplay between the p75^NTR^ and Trk receptors for neurotrophin signaling has been identified and occurs in several ways [15]. Hempstead et al. discovered that the co-expression of the Trk proto-oncogene and p75^NTR^ is required for the development of high-affinity NGF-binding sites [16]. Benedetti et al. discovered that a proper p75^NTR^ /Trk receptor ratio was essential for establishing high-affinity sites [17]. Bibel et al. further demonstrated the close proximity of p75^NTR^ and Trk receptors within cell membranes by immunoprecipitation in transfected cells and suggested that the signaling pathways they initiated would interact soon after their activation [15].

When neurotrophins bind to Trk receptors, they dimerize the receptor and autophosphorylate tyrosine residues, activating downstream signaling cascades [18]. The phosphatidylinositol 3 kinase (PI3K)/protein kinase B (Akt) signaling pathway, the Ras/mitogen-activated protein kinase (Ras/MAPK) signaling network, and the phospholipase C (PLC) signaling pathways are known to be activated or increased [9,19,20]. Some rapid signaling that controls a range of cellular activities, such as membrane excitability, synaptic transmission, and activity-dependent synaptic plasticity, is primarily mediated by Trk receptor interactions with ion channels and ionotropic receptors in the cell membrane [21].

p75^NTR^, the pan-neurotrophin receptor, plays various complex roles in regulating cell survival, neurodegeneration, and cell death. It initiates pro-apoptotic cascades through different signaling pathways [22], including the nuclear factor (NF)-κB pathway, the Jun kinase pathway, and the activity of Rho, which participate in neuronal survival, apoptosis, and growth cone motility, respectively [8,19]. While p75^NTR^ combines with sortilin, a co-receptor for neurotrophins, and forms heterodimers, apoptotic pathways can be activated [23,24]. Furthermore, p75^NTR^, in conjunction with sortilin, can increase the binding capability of proneurotrophins such as pro-NGF, a molecule involved in the death of oligodendrocytes, corticospinal neurons, and spinal motor neurons in the injured state [6,7,25,26]. As a result, the relative amount of pro-neurotrophins and mature neurotrophins or the number of Trk and p75^NTR^ receptors determines whether apoptotic or neurotrophic effects are generated [27].

The p75^NTR^ can also limit ligand-induced Trk receptor ubiquitination, postpone Trk receptor degradation, and therefore prolong the duration of Trk receptor-dependent signaling [28,29]. Additionally, p75^NTR^ can activate the Akt pathway, which plays an important role in the Trk-mediated neurotrophic signaling pathway, to support the pro-survival function, contrary to the popular hypothesis that p75^NTR^ is only responsible for neuronal cell apoptosis [4,30].

## 2. Neurotrophins and Traumatic Brain Injury

In response to the brain damage caused by traumatic brain injury (TBI), the brain can heal itself through a variety of compensatory processes known as neuroplasticity [31]. During post-traumatic brain remodeling processes, altered growth factor signaling, synaptogenesis, angiogenesis, neuron cell proliferation, gliogenesis, and cell structural changes can remodel the brain to improve functional recovery [32,33,34].

The choroid plexus reactively secretes neuropeptides that are consecutively distributed through the cerebrospinal fluid (CSF) to the traumatic region [35,36] to facilitate neurogenesis and parenchymal healing and restore cognitive ability [37,38,39,40]. Interestingly, how the signal transduction and homeostatic adjustment from a remote brain injury are transmitted to the choroid plexus, which is not directly destroyed by trauma, remains unknown. The modulating neuropeptides include CNTF, transforming growth factor β1 (TGF-β1) [41], fibroblast growth factor 2 (FGF2), epidermal growth factor (EGF), vascular endothelial growth factor (VEGF), NGF, IGF [36], GDNF [42,43,44], BDNF, and pituitary adenylate cyclase-activating polypeptide (PACAP) [45,46,47,48,49,50,51].

Studies of TBI and stroke have provided substantial evidence that neuropeptides, including neurotrophins, given exogenously to the CSF strengthen the protective effect and induce neuronal repair, regeneration, and synaptic sprouting. Numerous exogenously delivered CSF peptides, such as GDNF, PACAP, VEGF, IGF, FGF2, NGF, and BDNF, showed a significantly improved neurogenic capacity weakened in TBI [40,52,53]. For example, Sharma et al. found that the intracerebroventricular administration of BDNF, together with GDNF and IGF-1, significantly reduced the blood–brain barrier (BBB) and blood-CSF barrier (BCSFB) breakdown, cellular/tissue injuries, and brain edema formation in a rat model following whole-body hyperthermia, which causes the breakdown of the choroid plexus and was used to stimulate the mechanism of TBI in experiments [39].

Both human and murine studies have suggested that pro-neurotrophins are significantly upregulated following brain injuries or degenerative diseases, and the binding of these proteins to p75^NTR^ may control programmed neuronal cell death in injured or degenerative conditions [54,55,56]. The levels of endogenous neurotrophic factors are also affected by various kinds of CNS injuries, including TBI as well as other degenerative diseases [5]. An increase in their expression is thought to be one of the mechanisms for providing neuroprotection, neuronal repair, and neurogenesis following injury [57]. The expression of these growth factors is exemplified by NT-3, NT-4/5, NGF, BDNF, IGFs, bFGF, TGFs, and TNFs [58,59,60] (Table 1). Studies on these neuroprotective agents show that they reduce inflammation, free radical production, and cytoskeleton damage, whereas excitatory amino acids and inflammatory cytokines produced early in the secondary injury cascade disrupt intracellular calcium homeostasis [58]. Some studies also revealed that neurotrophins may serve as sensitive biomarkers for clinical outcomes of patients with TBI [61,62].

A number of recent investigations have indicated that the abnormalities in signaling pathways that are expected to have a role in many clinical conditions are important targets in neurotrophin signaling [63]. Because PI3K/AKT signaling is a powerful mechanism for inhibiting the activation of stress kinases and GSK-b, the stimulation of PI3K/AKT signaling by neurotrophin mimetics may provide neuroprotection [64]. For example, the flavonoid 7,8-dihydroxyflavone, a small TrkB agonist that mimics BDNF action, was found to have benefits comparable to those of BDNF in boosting neuronal survival and regeneration after TBI [65,66]. Because of its longer half-life and considerably smaller molecular size when compared to BDNF, it can cross the BBB; thus, it can be used in non-invasive clinical applications [67].

**Table 1:** Responses of neurotrophins after TBI.

Neurotrophins	Site	Level of Expression	Response	Remark/Note	Reference
NGF	Hippocampus in rats	Protein	Biphasic	Increase: 6 h	[68]
				Decline: 7 days	
				Second rise: 14 days	
	hippocampus in rats with CSF dissemination	Protein	Increase	None	[68]
BDNF	Hippocampus in rats	mRNA of BDNF and mRNA of TrkB	Increase	None	[69]
	Cortex ipsilateral to the lesion site and the bilateral dorsal hippocampus in rats	mRNA	Increase	None	[70]
	Hippocampus ipsilateral to the lesion site in rats	mRNA	Decrease	None	[71]
	Hippocampus contralateral to the lesion site in rats	mRNA	Increase	Sustained for 2 weeks	[71]
		Protein	Decrease	Post-injury 24 h	[72]
			Same as post-TBI baseline	Post-injury 36 h	[72]
	Hippocampus and dentate gyrus in rats	mRNA of BDNF and mRNA of TrkB	Increase	None	[73]
NT-3	Hippocampus in rats	mRNA	Decrease	None	[69]
	Dentate gyrus and cornu ammonis 2 regions of the hippocampus in rats	mRNA and protein	Decrease	In the first 12–24 h following TBI	[74,75]
	Rat brain	mRNA	Same as post-TBI baseline	None	[70]
NT-4/5	Injured cortex and hippocampus in rats	Protein	Increase	In the acute period (within 3 days)	[60]

### 2.1. Nerve Growth Factor

In the early 1950s, Levi-Montalcini revealed that NGF may control the survival and maturation of developing PNS neurons [3]. Since then, NGF has become one of the most well-known members of the neurotrophin family for promoting axonal sprouting, dendritic formation, and cell body expansion in neural systems, and studies about relevant therapeutics also emerged afterwards [76,77,78].

In response to TBI, NGF in the hippocampus is quickly upregulated and exhibits a biphasic response. In a controlled cortical impact damage model in rats, DeKosky et al. found that NGF increased at 6 h, dropped at 7 days, and then increased again at 14 days [68]. They also showed that NGF participated in initializing post-TBI antioxidant reactions in rats by activating several antioxidant enzymes, including the upregulation of catalase (CAT), glutathione peroxidase (GPx), and the downregulation of superoxide dismutase (SOD) [68]. Dixon et al. concluded that post-TBI NGF elevation in the hippocampus and its dissemination through CSF help restore cholinergic neurotransmission and cognitive function, particularly spatial memory, in a rat TBI model generated using a controlled cortical impact injury device [79]. Hicks et al. discovered that a mild lateral fluid percussion brain injury in rats induced significant increases in BDNF and TrkB mRNAs as well as a decrease in NT-3 mRNA, demonstrating that even mild TBI differentially alters neurotrophin and neurotrophin receptor levels in the hippocampus [69].

Clinically, NGF concentrations in the CSF have a significant correlation with the severity of TBI and can serve as a biomarker for predicting neurological outcomes. Chiaretti et al. conducted an observational study indicating that higher NGF and lower interleukin (IL) 1β concentrations at 2 h after TBI correlated significantly with favorable neurological outcomes in children with TBI, whereas no outcome correlation was found for IL-6, BDNF, or GDNF expression [61]. Meanwhile, NGF may be able to trigger inflammatory cascades with negative consequences on surrounding tissues because NGF levels are increased in many inflammatory disorders such as chronic arthritis and multiple sclerosis [80,81]. NGF is well-known for its complex involvement in nociceptive processing [82]. Dai et al. discovered in a rat model of neuropathic pain that inhibiting TAK1-MAPK-/NF-κB signaling in the periphery reduced neuropathic pain [83]. Despite the abundance of literature on NGF-mediated neuropathic pain modulation, the specific signaling pathways downstream of NGF receptor activation that produce nociception are complex and poorly understood [82].

### 2.2. Brain-Derived Neurotrophic Factor

BDNF is the most abundant neurotrophin in the CNS [84] and the most widely studied, owing to its potential effects and wide distribution in the brain [85]. The coordination of the BDNF and TrkB receptors governs a wide range of neural activities, including neuronal survival, differentiation, migration, axonal sprouting, synaptogenesis, plasticity, and the enhancement of long-term potentiation (LTP), which influences learning and memory [86,87,88]. Growing studies focusing on BDNF have emerged, but there are discrepancies among the results of BDNF expression at different post-injury times, regions in the CNS, genetic polymorphisms, and clinical outcomes [89,90,91].

#### 2.2.1. Divergent Expression of BDNF

In terms of BDNF expression in various CNS areas, Yang et al. discovered that BDNF mRNA expression was considerably enhanced in the cortex ipsilateral to the injured side and bilateral dorsal hippocampus in a rat model following TBI caused by a controlled lateral cortical impact [70]. In another rat model, however, following a TBI caused by the controlled penetration of a 2-mm-thick needle-shaped item, Rostami et al. found that BDNF mRNA expression was reduced in the hippocampus ipsilateral to the lesion, but it was increased on the contralateral side [71]. As for discrepancies in post-injury time course, the level of BDNF was significantly down-regulated at 24 h post-injury and returned to the level with no significant difference at 36 h post-injury compared with 0 h post-injury [72]. Nonetheless, Rostami et al. discovered that the increase in BDNF mRNA expression in the hippocampus lasted for 2 weeks following TBI [71]. Merlio et al. demonstrated that the mRNA expression level of the TrkB receptor is similarly upregulated in the same time course with BDNF in the hippocampus and dentate gyrus from a rat model with electrical stimulation with a rapid kindling paradigm in the hippocampus [73]. The surge of BDNF and its corresponding receptor indicated that BDNF has the capacity to attenuate secondary cell injuries and protect the neuronal function following TBI [5]. Distinct regulatory signaling cascades between cortisol and BDNF may influence secondary injury mechanisms after TBI and predict clinical outcomes. Munoz et al. conducted a prospective cohort study with 117 patients and discovered that BDNF in the CSF is related to patients’ cortisol levels, which can strongly predict a 6-month mortality after severe TBI. These findings suggest that hormone and neurotrophin levels may be related to the degree of neurological damage [62].

#### 2.2.2. Single-Nucleotide Polymorphism of BDNF

The BDNF protein possesses a single-nucleotide polymorphism (SNP) site at rs6265, at the 66-amino acid position, which results in the replacement of wildtype Val with Met (Val66Met, valine to methionine) [92]. Although other SNPs of BDNF such as rs71244, rs1519480, and rs1153659 have been reported to influence the outcome after TBI, the most studied polymorphism of BDNF in humans is rs6265 (Val66Met) [89]. The frequency of the Met66 allele is approximately 25–32% in Caucasian populations and 40–50% in Asian populations [93]. Egan et al. found that when Val66 (vBDNF) in the BDNF sequence is substituted with a Met (mBDNF), the depolarization-dependent BDNF production in human hippocampal neurons is severely reduced [94]. Consequently, mBDNF tends to accumulate in the soma, whereas vBDNF accumulates in punctate vesicles in the dendrites. Neurons secrete vBDNF but not mBDNF in response to activity. The activity-dependent release of BDNF drives LTP [95]; thus, Val66Met SNP carriers have impaired neuroplasticity [94].

Giarratana et al. discovered that injured Val66Met carriers had a larger inflammatory volume, increased apoptosis, and gliosis at 1 day or 21 days post-TBI in a mouse model following repeated mild TBI utilizing a lateral fluid percussion technique. In terms of apoptosis-inducing pro-BDNF (precursor) and survival-inducing mature BDNF levels in the hippocampus, the Val66Met carriers had a lower total BDNF and a higher pro/mature ratio of BDNF than the wild-type Val66Val carriers. According to animal research, the Val66Met SNP is a risk factor for poor outcomes after mild TBI [92].

As to the influence of the Val66Met SNP in mild TBI human cases, Wang et al. found more depression and anxiety in Val66Met carriers at 1 and 6 weeks post-TBI from a 192-cases cohort [96].

However, in severe TBI cases from phase 3 of the Vietnam Head Injury Study (VHIS) registry, the Val66Met polymorphism partially helped the neurological adaptation and promoted the recovery of executive functions such as intelligence, memory, and processing speed in patients with severe TBI [97,98]. It is worth mentioning that the brain function was assessed in these veterans at very long-term time points, up to more than 40 years after TBI. Val66Met carriers secrete less mature BDNF and proBDNF (the precursor of BDNF) in response to activity. mBDNF is protective after severe TBI, suggesting that the apoptotic action of proBDNF overcomes the survival signaling of mature BDNF in this context [90].

### 2.3. Neurotrophin 3

NT-3 is a neurotrophin family member with a remarkable number of amino acid identities and four variable domains that are structurally linked to other neurotrophins such as NGF and BDNF [99,100]. NT-3, a trophic factor for sympathetic and sensory neurons [101], regulates neuronal survival, differentiation, and maintenance in the PNS and CNS [102,103,104,105]. NT-3 exerts functional effects by attaching to the TrkC receptor with high affinity [106].

Endogenous NT-3 protein levels and NT-3 mRNA expression are considerably lower in the first 12–24 h after TBI [107,108], including the dentate gyrus [74] and cornu ammonis (CA) 2 region [75] of the hippocampus, indicating a negative function of NT-3 in the early phase of injury [109]. Endogenous NT-3, according to Bates et al., accelerates neuronal death in cortical cells induced by oxygen-glucose deprivation, potentially by increasing reactive oxygen species [110]. The downregulation of NT-3 is most likely a protective mechanism.

However, reverse results have been reported in some rat models. One study showed that TBI proceeded without changes in NT-3 mRNA expression [70], whereas another study showed that, in corticospinal neurons, exogenous NT-3 significantly prevented the axotomy-induced death of neuronal cells, showing the protective and beneficial roles of NT-3 [111]. Furthermore, in rat models, the topical administration of NT-3 decreased brain edema and BBB permeability while improving the consciousness level following TBI [107].

As for studies focused on human diseases in the CNS, recombinant NT-3 administered in a specific regimen has been proven to be safe without serious complications in human bodies [109,112]. A preliminary clinical trial demonstrated the potential for the subcutaneous injection of NT-3 in patients with CMT1A, a PNS neuropathy caused by a mutation in the gene-encoding peripheral myelin protein in Schwann cells [113]. Although these data indicate the therapeutic use of NT-3, larger cohort studies are needed before it can be applied to TBI.

### 2.4. Neurotrophin 4/5

NT-4 is a member of the neurotrophin family that regulates the survival, differentiation, and regeneration of mammalian neurons. It is also known as NT-5 [114,115,116]. NT-4/5, the least understood neurotrophin, is the most widely distributed neurotrophin that exists universally in embryonic and adult rat tissues and appears to be less affected by environmental signals in surrounding regions in contrast to other neurotrophins [117].

NT-4/5 can relieve the neuroinflammation and enhance the neurological functioning in newborn rat models with germinal matrix hemorrhage (GMH) via the TrkB/PI3K/FoxO1 pathway and may be a potential treatment for neuroinflammation and hydrocephalus following GMH or other comparable brain lesions [118]. NT-4/5 might protect a cultured embryonic rat hippocampus and cortical neurons from glucose deprivation-induced damage as well as decrease neuronal vulnerability to glutamate toxicity [119,120]. Furthermore, neurons treated with NT-4/5 were more resistant to toxicity caused by calcium ion carriers, showing that NT-4/5 conferred neuronal resistance to calcium-mediated damage [119].

NT-4/5-deficient mice exhibited preferential pyramidal cell loss in the CA of the hippocampus following TBI as well as a prolonged recovery period of motor function compared with their brain-injured controls [60]. The loss of pyramidal cells in CA could be restored with early and sustained infusions of recombinant NT-4/5, but not with functional impairment [121], indicating that NT-4/5 may serve as an adaptive neuroprotective response in the damaged neuronal network [60]. Endogenous NT-4/5 expression and release, which were shown to be upregulated following TBI, are modulated by CNS disorders such as TBI [59,60], particularly in the damaged cortex and hippocampus in the acute phase (3 days) [60]. In addition, NT-4/5 secreted at inflammation sites is thought to be useful in tissue regeneration and shows potential as a future treatment option after TBI [60,121]. As a result, NT-4/5 plays a key role in brain development and has the capacity to recapitulate numerous processes involved in brain growth as well as providing neuroprotection for susceptible neurons [58].

## 3. Potential Therapeutic Role of Neurotrophins in TBI

### 3.1. Diet

Several studies have emphasized the vital role of nutrition in influencing TBI prognosis and recommended various beneficial diets to the patients with TBI via regulating neurotrophins and/or their receptors, including procyanidins [122], vitamin E supplements [123], blueberry [124], docosahexaenoic acid (DHA) supplement [125], omega-3 fatty acids [126,127], ethanol intoxication [128], curcumin [129], chronic caloric restriction [130], astaxanthin [131], dl-3-*n*-butylphthalide (NBP) [132], cysteine-rich whey protein supplement (Immunocal^®^) [133], resolvin D1 (RvD1) [134], and trehalose [135]. Conversely, there is still a dietary category that aggravates the impairment in TBI patients, including high-saturated-fat diets [123].

Procyanidins, with a potent antioxidant activity, were hypothesized to engage in TBI neuroprotection by lowering malondialdehyde levels and increasing glutathione (GSH) and SOD levels. Furthermore, procyanidins increased the BDNF, cyclic AMP (cAMP), and cAMP response element binding protein (CREB) levels, which resulted in a better cognitive performance in the Morris water maze [122].

In a rat model of fluid percussion injury, vitamin E supplementation can protect against cognitive impairment after TBI by engaging in BDNF-mediated synaptic plasticity, that is, synapsin I, CREB, and calcium/calmodulin-dependent protein kinase II [136].

Blueberry consumption shortly after TBI reduces behavioral impairment and neural dysfunction. Blueberry food supplementation improves spatial and object recognition memory and increases BDNF-mediated plasticity in a rat model of fluid percussion injury [124]. Furthermore, a human study revealed that the consumption of coffee berries also induced increased levels of plasma BDNF by 143%, compared with baseline. Therefore, coffee berries might have a BDNF-dependent effect in TBI. However, there is no study directly clarifying the effects of coffee berries in TBI [137].

DHA in the diet may protect against learning impairment after TBI by increasing BDNF-mediated plasticity in a fluid percussion injury rat model [125].

Sufficient dietary omega-3 fatty acid supplementation was associated with increased levels of BDNF, TrkB receptor, and CREB expression in the lumbar spinal cord and acquired resistance to energy homeostasis changes and mitochondrial metabolic imbalance in a rat model of fluid percussion injury [126,127].

Ethanol can only reverse the injury-induced responses and downregulate the regulatory expression of the BDNF gene merely in moderate TBI with ethanol pre-TBI treatment in a closed weight-drop TBI mouse model [128].

Curcumin is an effective free radical scavenger [138]. Wu et al. demonstrated that consuming the antioxidant curcumin can decrease the negative effects of TBI on synaptic plasticity and cognition by restoring altered levels of BDNF, synapsin I, and CREB following TBI in a mild fluid percussion injury rat model of TBI [129].

In a controlled cortical impact rat model, chronic calorie restriction reduced the extent of the cortical lesion after damage, improved spatial memory, and increased BDNF levels in the cortical area surrounding the site of injury and in the hippocampus [130].

Compared with the vehicle-treated TBI group in the M.A. Flierl weight-drop mouse TBI model, an astaxanthin treatment through oral gavage improved the sensorimotor performance, improved the cognitive function recovery and reduced the lesion size and neuronal death in the cortex. Astaxanthin also restored the levels of BDNF, growth-associated protein 43, synapsin, and synaptophysin in the cerebral cortex, indicating that it promotes neuronal survival and plasticity [131].

NBP therapy enhanced the sensorimotor functional recovery and substantially decreased the post-TBI depressed behavior. A consecutive daily intranasal NBP administration enhanced neurogenesis, angiogenesis, and arteriogenesis in the post-TBI brain in a controlled cortical impact mouse model, followed by the upregulations of BDNF, VEGF, endothelial-derived nitric oxide synthase (eNOS), and matrix metallopeptidase 9 (MMP-9) [132].

Immunocal^®^ is a non-denatured whey protein product that has been demonstrated to work as a cysteine delivery method to enhance GSH levels [139]. In the controlled cortical impact mouse TBI model, there was a significant preservation of axonal myelination, a significant decrease in degenerating neurons, a reduction in Iba1 (microglial marker), and a preservation of BDNF in the brains of Immunocal^®^-pretreated mice compared with untreated TBI mice, indicating that Immunocal^®^ supplementation before TBI significantly improves the resilience to TBI [133].

In a controlled cortical impact mouse TBI model, RvD1 was found to enhance the expression of glutamate aspartate transporter and BDNF in the hippocampus. By regulating neuroinflammation and preserving astrocytic mitochondria, RvD1 may be a promising treatment approach for reversing cognitive impairment after TBI [134].

In a controlled cortical impact injury mouse TBI model, trehalose therapy resulted in a substantial increase in BDNF and pro-BDNF expression in the contralateral cortex, correlating with both synaptophysin and doublecortin protein expression levels in the same area. These findings suggest that trehalose has a neuroprotective effect in TBI and may also play a role in post-injury synaptic reorganization [135].

### 3.2. Exercise

Apart from diet, exercise also has beneficial but selective effects on the human cognitive function, especially in older adults [140,141]. The application of both physical activity and diet have variable effects [142]. Among all the effects published, BDNF seemed to be involved in the mechanism of exercise-associated regulation; hence, exercise was thought to be the potential factor for neural recovery in patients with TBI [143,144].

In all of the TBI animal models studied, nine studies found that exercise increased BDNF levels and/or BDNF mRNA expression, showing that the activation of BDNF-TrkB receptors is crucial in the mechanism of exercise-modulated effects [145,146,147,148,149,150,151,152,153]. However, one study found that exercise regimens with significant stress reactions may not be helpful during the early post-injury phase, resulting in elevated BDNF levels in exercise-treated fluid percussion injury rat TBI models [154].

Interestingly, five studies followed the previous ones and tried to modulate some of the parameters in experiments such as voluntary or forced exercise, the onset time of exercise, and the intensity of exercises. These three parameters all modulated some of the variables to determine whether the dependent effects of exercise on TBI-induced impairments existed. One study showed that forced exercise led to decreased BDNF levels, whereas voluntary exercise increased the BDNF levels. Since forced physical training was seen as a stressor that elevated the corticosterone levels [154], it seemed that strong stressors would retard the repair and recovery from TBI destruction [155]. The intensity of exercise was also the focus. The results demonstrated that a low-intensity exercise after TBI is effective in accelerating the recovery of TBI and upregulating the BDNF levels rather than a high-intensity exercise [156]. The onset time of exercise significantly affects the outcome of animals with TBI. Chen et al. demonstrated in a closed-head-injury mice model that an early exercise protocol (2 days post-injury) drastically increased the ability of recognition and memory, extensively protected the neurons from death, and promoted neurite regeneration compared with the late exercise protocol (9 days post-injury) [157]. In contrast, a further restoration of BDNF reduction induced by TBI was presented on the group with late exercises. This indicated that the restoration of BDNF would be disrupted in the closed-head-injury mouse model if the exercise intervention was administered too early [157]. Deductively, different severities of TBI need to match different onset times of exercise to produce maximal therapeutic effects [158].

Exercise benefits animals with TBI not only post-traumatically but also pre-traumatically. Studies of preventive exercise before TBI events showed increased BDNF concentrations, enhanced sensorimotor performance and cognitive functions, and decreased lesion volume in animals with exercise training before TBI [152,153].

### 3.3. Stem Cell Therapy

Because exogenous neurotrophins have difficulties crossing the BBB, innovative treatments such as mesenchymal stem cells, which have the unique ability to self-renew and differentiate into several lineages, have been proposed [159] (Table 2). According to previous studies, neural mesenchymal stem cells differentiate into new neurons as well as neuroglia and further modulate the production of neurotrophins in specific regions as well as the reaction of neural stem/stromal cells in response to CNS-related diseases or injuries, including TBI [160,161]. Nevertheless, there are still limitations such as the precise modulation of differentiation, ethical problems regarding the sources of stem cells, graft-versus-host diseases, and the safety and effectiveness of stem cell therapy [162].

#### 3.3.1. Bone Marrow Stem Cells

Studies on rat TBI models treated with bone marrow stem cells (BMSCs) in response to TBI have yielded substantial positive results [164]. Rat TBI models administered with BMSCs generally presented better neurological outcomes with improved cognitive functions, behavioral performance, and shorter latency to reaction in the tests [165]. The neuronal loss and impairment induced by TBI were decreased, whereas the transplantation of these cells migrated into or around the injured areas, where more differentiated neurons and astrocytes were observed immunologically [163]. In terms of neurotrophins, increased NGF [163,164] and BDNF [163,164,165,166] expression was found in the CNS of the animals, whereas the decreased expression of the BCL2-associated agonist of cell death and BCL2-associated X protein signaling and increased synaptophysin explained the possible mechanism involved in the stem cell therapy process. A study on *NT-3^P75−2^* gene-modified BMSCs, which overexpress NT-3 proteins, also showed satisfactory outcomes in vitro and in vivo following TBI [167].

#### 3.3.2. Human Mesenchymal Stem Cells

Animals treated with human mesenchymal stem cells (hMSCs) following TBI showed an improved neurological function, cognitive capacity, and memory capacity, decreased volume of lesion areas, and attenuated brain edema. hMSCs are applied along with Wharton’s jelly, which is primarily composed of mucopolysaccharides and a matrix from which hMSCs can be extracted [168,169,170]. BDNF, NGF, and NT-3 were significantly elevated at 2 days post-injury [169], whereas in another study the increase in BDNF mRNA expression was also found at 14 days post-injury [168]. The results of a western blot analysis of up-regulated Akt expression and decreased caspase 3 cleavage after TBI also presented a possible mechanism underlying hMSCs therapy in TBI [169]. However, evidence-based effective methods of delivery, such as intraperitoneal administration [168], are also critical for efficiently correcting TBI damage, since the subarachnoid injection of hMSCs in TBI rats exhibited no significant changes in their neurological performance and neurotrophin expression [171]. Bonilla discovered that subarachnoid-transplanted cells were localized near the injection site. Some were identified at the site of the lesion, but no mesenchymal stem cells were detected in the healthy brain tissue. This might be the reason why the subarachnoid injection caused no significant difference [171]. When combined with pioglitazone, which reduces the pro-inflammatory chemokines, the hMSCs further showed enhanced sensorimotor responses and increased BDNF expression [172].

#### 3.3.3. Neural Stem Cells

Apart from the stem cells mentioned above, neural stem cells (NSCs) have shown neuroprotective properties in response to TBI [173]. Mice injected with NSCs after TBI showed improved spatial and memory functions in addition to the enhanced neurological functions [174]. Morphologically, the NSCs successfully survived in the hippocampus of transplanted rats, especially in the CA1 region [184], and largely differentiated into neurons with anti-inflammatory functions with surrounding astrocytes. In terms of neurotrophins and neurotrophic factors, NSC treatment enhanced the expression of NGF, BDNF, NT-3, GDNF, cerebral dopamine neurotrophic factor [174], and synaptophysin [173]. The mechanism underlying the phenomenon might lie in the decreased expression of glutamic acid and α-smooth muscle actin [185] and the maintained level of γ-aminobutyric acid, indicating the downregulated excitotoxicity of neurotransmitters [184]. However, the intensity of trauma is still important because the up-regulation of neurotrophins in NSC treatment was significant only in the condition of mild TBI instead of severe TBI [175]. The gene-modified NSCs overexpressing BDNF showed a better rate of survival and differentiation [176,177]. Nevertheless, an artificial gene-modified NSC overexpressing multineurotrophin (MNTS1), a neurotrophin binding and activating all three types of Trk receptors, showed no significant improvement in the cytoarchitecture and behavioral ability compared with the non-genetically modified NCS-treated mice, indicating the superfluous augmentation of NSC-MNTS1 [186].

#### 3.3.4. Human Umbilical Cord Mesenchymal Stem Cells

Another subpopulation known as human umbilical cord mesenchymal stem cells (hUC-MSCs) was also tested for its potential usage in the treatment of TBI. Irrespective of whether umbilical cords were derived from neonatal rats [178], contained in chitosan scaffolds [179], or extracted from a human umbilical cord-matrix [180], the animals given hUC-MSCs showed better neurological severity scores and alleviated brain edema after TBI compared to the mice without stem cell treatment. Morphologically, the promotion of neural regeneration, improved rates of differentiation, and inhibition of inflammatory cells have also been well demonstrated. The expressions of BDNF, GDNF [178], glial fibrillary acidic protein, and microvessel density were significantly upregulated in the CNS of hUC-MSCs-treated mice [180]. NGF and associated TrkA receptors as well as p75^NTR^ were also highly expressed in similar studies that treated mice with human umbilical cord blood CD45^+^ cell cultures [181]. Perivascular cells form a human umbilical cord have been shown to prevent axonal degeneration in response to NGF withdrawal or oxygen glucose deprivation in vitro [182]. Furthermore, a co-culture of hUC-MSCs and activated astrocytes as well as forcing hUC-MSCs with CXC chemokine receptor 4 [183], which may stimulate stem cell migration, improved the differentiation and repair capacities in the brain parenchyma.

## 4. Energy Balance Regulation by Neurotrophins in TBI

According to the metabolite availability, the brain is an energy-intensive organ that may use glucose or ketone bodies as energy sources [187]. The rise in glutamate levels in TBI commences a few minutes after the initial trauma, peaks in approximately 10 minutes, and lasts for many days [188]. The elevated release of glutamate into the extracellular milieu following injury is a pivotal event that causes metabolic dysfunction, generating significant increases in cerebral glucose utilization and extracellular lactate production [189,190,191,192,193]. Mitochondrial dysfunction is another significant event associated with the post-TBI energy crisis, since the increased energetic demand cannot be satisfied [194] Furthermore, following TBI, the glucose metabolism is changed, which is attributed in part to oxygen deprivation [195]. After TBI, there is a rise in the Lactate/Pyruvate ratio, indicating a shift toward anaerobic metabolism in both animal models [196,197] and human beings [198]. The deregulation of the brain’s metabolism results in a decrease in cerebral energy generation. Reduced ATP levels then induce the failure of ATP-dependent ion channels and proteins, resulting in ionic osmotic changes that cause cell swelling and eventually cell death [199]. As a result, the restoration of injured nervous tissue necessitates more energy than the tissue’s normal physiological condition [200].

Some studies indicate that BDNF signaling in the brain mediates the beneficial impacts of a cognitively and physically demanding environment on energy metabolism, such as enhanced insulin sensitivity [201], increased brown fat genesis [202,203], and cardiovascular fitness [204]. Inhibiting BDNF/TrkB signaling in the periphery resulted in the down-regulation of numerous metabolic factors, including AMP-activated protein kinase (AMPK), a protein kinase associated with cell energy regulation homeostasis [205]. BDNF may give a survival advantage to individuals experiencing nervous system damage by boosting mitochondrial biogenesis and increasing neuronal resilience to injury by up-regulating the expression of genes encoding cytoprotective proteins [206].

Several neurotrophins have emerged as key participants in the complicated systems that control food intake and energy expenditure as well as in potential disease processes that contribute to obesity [207]. The molecular and cellular foundations of the neurotrophin function affecting the energy balance and cerebral metabolism in TBI are crucial research topics that need to be explored further [187].

## 5. Conclusions

Neurotrophins play important roles in many aspects of neural development, survival, and plasticity. Many studies have focused on the response of neurotrophins during TBI. Some possible therapeutic effects of neurotrophins in TBI have also been demonstrated. By summarizing these studies, we hope to provide some insights for further research in the field of neurotrophins and TBI.

## Figures and Tables

**Table 2:** Potential therapeutic roles of neurotrophins in stem cell therapy for TBI.

Stem Cell Type	Stem Cell Source	Model	Key Neurotrophin	Route	Description	Reference
BMSC	SD rat	SD rat	NGF, BDNF	Injured brain site transplant	TBI healing can be aided by BMSCs with SDF-1-induced CXCR4 expression.	[163]
BMSC	Wistar rat	Wistar rat	NGF, BDNF	Intravenous	BMSCs contribute to the improvement of the functional outcome of TBI rats.	[164]
BMSC	SD rat	SD rat	BDNF	Intravenous	BMSCs significantly reduce TBI-induced neuromotor impairment and neuronal loss.	[165]
BMSC	Wistar rat	Wistar rat	NGF, BDNF	Intravenous	BMSC treatment promotes functional recovery. BMSCs induce growth factor production.	[166]
BMSC	SD rat	Mice	NT-3, NT-3^P75−2^	Injured brain site transplant	In a mouse TBI model, *NT-3^P75−2^*-gene-modified bone mesenchymal stem cells enhance neurological function recovery.	[167]
hUC-MSCs	WJ tissue from hUC	SD rat	BDNF	Injured brain site transplant	In a rat model of TBI, WJ transplantation improves brain function.	[168]
hMSC	Human	SD rat	NGF, BDNF, NT-3	Injured brain site transplant	TBI treated with hMSCs in the acute period can improve neurological the functional outcome.	[169]
MSC	Rat	SD rat	BDNF	Intravenous	After TBI, BDNF-induced MSCs-Exo may successfully enhance functional recovery and neurogenesis in rats.	[170]
MSC	Wistar rat	Wistar rat	NGF, BDNF	Injured brain site transplant	There are differences in neurotrophin expression, although they are not statistically significant.	[171]
hMSC	Human	SD rat	BDNF	Intravenous	Reducing proinflammatory cytokine expression in the brain tissues after TBI and before hMSC therapy enhances the success of the therapy, in which BDNF may have a role.	[172]
NSC	GFP+ C57BL/6 mice	SD rat	BDNF	Injured brain site transplant	TBI functional recovery is aided by NSC transplantation via BDNF-mediated neuroplasticity.	[173]
AMSC, AM-NSC	Human	SD rat	NGF, BDNF, NT-3	Injured brain site transplant	TBI in rats can be effectively treated using neural stem-like cells generated from human amnion tissue.	[174]
Differentiation ESC	Mouse ES cell line	C57BL/6 mice	NGF, BDNF	Injured brain site transplant	The transplanted neurospheres were able to survive in the mild TBI mice but not in the severe TBI animals.	[175]
NSC	Wistar rat	Wistar rat	BDNF	Injured brain site transplant	The protective impact of BDNF-modified NSCs transplantation outperforms that of naive NSCs transplantation.	[176]
NSC	Wistar rat	Wistar rat	BDNF	Injured brain site transplant	Following NSC transplantation, BDNF enhances synaptic protein levels via the MAPK/Erk signaling pathway and the Nrf2/Trx axis in a rat model of TBI.	[177]
UC-MSC	Human	SD rat	BDNF	Injured brain site transplant	By inhibiting the release of inflammatory factors and increasing the production of GDNF and BDNF, UC-MSCs may play an essential role in TBI recovery.	[178]
NSC, hUC-MSC	SD rat, human	SD rat	BDNF	Injured brain site transplant	CGB scaffolds coated with hUC-MSCs can have two impacts for TBI treatment: they can compensate for neuron loss after TBI and they can release active BDNF from the scaffold, leading NSCs in situ in the brain to develop into neurons.	[179]
hUC-MSC	Human	SD rat	BDNF	Injured brain site transplant	Transplantation of UC-MSCs for the treatment of acute TBI can significantly decrease damage and enhance vascular repair.	[180]
hUC-MSC	Human	C57BL/6 mice	NGF	Intravenous	This work shows that NGF-induced anti-inflammatory and immunomodulatory characteristics of the CD45+ subpopulation are mediated via systemic IV xenotransplantation into TBI mice.	[181]
HUCPVC	Human	SD rat	NGF, NT-3	Intravenous	This work demonstrates the importance of perivascular cells in shielding axons from damage and suggests a possible cell-based treatment to treat secondary injury after TBI.	[182]
hUC-MSC	Human	SD rat	BDNF	Injured brain site transplant	This study discovered that the co-culture of hUC-MSCs and activated astrocytes increases BDNF production, which may enhance both ectogenic hUC-MSC neural development and endogenic neurogenesis.	[183]

AM-NSC; AMSC; BDNF, brain-derived neurotrophic factor; BMSC, bone marrow stem cell; CGB, genipin-crosslinked; CXCR4, CXC chemokine receptor 4; ES; ESC; GDNF, glial cell line-derived neurotrophic factor; hMSC, human mesenchymal stem cell; hUC, human umbilical cord; HUCPVC, human umbilical cord perivascular cells; MAPK, mitogen-activated protein kinase; MSC, mesenchymal stem cell; NGF, nerve growth factor; Nrf2, nuclear factor erythroid 2-related factor 2; NSC, neural stem cell; NT-3, neurotrophin 3; SD, Sprague–Dawley; SDF-1, stromal cell-derived factor 1; TBI, traumatic brain injury; Trx, thioredoxin-1; UC-MSC, umbilical cord mesenchymal stem cell; WJ, Wharton’s jelly.

## Data Availability

Not applicable.
